# Target Trial Emulation and the TARGET Guideline to Advance Rural and Remote Health Research

**DOI:** 10.5694/mja2.70205

**Published:** 2026-05-14

**Authors:** Tanvir Kapoor, Harrison J. Hansford, Brooke A. Spaeth, Adam D. Irwin, Aidan G. Cashin

**Affiliations:** ^1^ Jandowae Multipurpose Health Service Darling Downs Hospital and Health Service Jandowae Queensland Australia; ^2^ School of Medicine, Faculty of Health Griffith University Gold Coast Queensland Australia; ^3^ School of Health Sciences UNSW Sydney Sydney New South Wales Australia; ^4^ Centre for Pain IMPACT Neuroscience Research Australia Sydney New South Wales Australia; ^5^ International Centre for Point‐Of‐Care Testing Flinders University Adelaide South Australia Australia; ^6^ Health Equity Impact Program Flinders Health and Medical Research Institute Adelaide South Australia Australia; ^7^ Centre for Clinical Research University of Queensland Brisbane Queensland Australia; ^8^ Infectious Diseases Queensland Children's Hospital Brisbane Queensland Australia; ^9^ School of Population Health, Faculty of Medicine UNSW Sydney Sydney New South Wales Australia

**Keywords:** guidelines as topic, health systems, rural health services

## Abstract

Rural and remote Australians experience persistent health inequities. Although randomised controlled trials (RCTs) remain the most rigorous method for establishing causal relationships and informing equitable health policy, they are challenging to conduct in rural settings, contributing to under‐representation in research. When RCTs are not feasible, observational analyses using the target trial framework provide a rigorous and pragmatic alternative. Despite growing international adoption, this approach has not been applied to rural contexts. The recently published TARGET guideline offers transparent reporting standards for these studies, providing a new methodological tool to advance rural and remote health research.

## 1 Introduction

Rural and remote Australians experience poorer health outcomes, reduced access to timely care and shorter life expectancy than those living in metropolitan areas [1]. Despite decades of investment in service innovation, much of rural health policy still relies on evidence generated in urban or international contexts [2, 3]. Enabling clinical trial activity in these areas is an important step towards improving evidence relevance, strengthening local research capacity and supporting implementation of effective system changes [2]. However, although randomised controlled trials (RCTs) remain the most rigorous means of establishing causal relationships, they are uniquely challenging in rural settings because of small populations, logistical complexity and ethical concerns about withholding interventions from high‐need communities [4, 5, 6, 7, 8].

Using observational data, target trial emulation offers a pragmatic approach when randomised trials are not feasible [9]. By specifying a hypothetical RCT and mapping this design to the observational data, target trial emulation can improve the design of observational studies to balance rigour and real‐world feasibility [10, 11]. This approach facilitates credible, transparent and timely evaluation of system‐level interventions that may be challenging to randomise, such as workforce programmes, telehealth initiatives or point‐of‐care diagnostic testing [10].

This perspective article outlines how target trial emulation can strengthen rural health research by providing a structured framework for causal inference when RCTs are impractical. It illustrates this approach with an example uniquely relevant to rural health systems and concludes by highlighting the recently published TARGET guideline (Transparent Reporting of Observational Studies Emulating a Target Trial), which sets international standards for transparent reporting of target trial emulation [12].

## 2 The Promise of Target Trial Emulation in Rural Research

Target trial emulation is a conceptual framework that aims to improve the design and reporting of observational studies [9]. It involves explicitly defining the key elements of a hypothetical RCT (i.e., eligibility criteria, interventions and comparators, assignment procedures, outcomes, follow‐up, causal contrasts, assumptions and data analysis plan) and then emulating (mapping) that design with observational data [13]. This framework mitigates key design‐related sources of bias that are common in observational studies, including immortal‐time bias and misclassification of treatment strategies [14, 15]. Researchers can then focus their attention on addressing important data‐related biases (e.g., confounding) through appropriate data collection and analytical methods such as inverse‐probability weighting and marginal structural models in an attempt to generate similar patient cohorts, as would be expected under randomisation [11]. When benchmarked against RCTs, target trial emulations have been shown to yield comparable estimates across a range of interventions and populations [16, 17, 18, 19]. This convergence demonstrates the potential to generate reliable evidence in observational emulations of target trials when randomisation may be infeasible or unethical.

With the increasing availability of real‐world data routinely collected through administrative databases, target trial emulation enables researchers to assess the effectiveness of interventions under conditions that are representative of everyday clinical practice, providing evidence that is both timely and policy‐relevant [20, 21]. For rural health systems, where RCTs are under‐represented, this capacity for robust, pragmatic and context‐specific observational evaluation is particularly valuable.

Many small health facilities, for example, lack the dedicated governance and reporting infrastructure required to host RCTs [8]. Even trials designed to minimise site burden through waivers of consent or passive data collection can experience lengthy delays due to these constraints, impeding progress by months or even years [4, 8].

Target trial emulation should not replace RCTs, but it offers a complementary pathway to overcome these barriers. By drawing on real‐world data, this approach may reduce strain on rural research infrastructure while maintaining methodological rigour. Although it does not expand active participation in the way randomised trials do, target trial emulation broadens access to evidence generation, which supports the inclusion of rural populations in high‐quality causal analyses and highlights areas where randomised trials are needed. This reframing aligns with the Medical Research Future Fund's Measures of Success, particularly the goal of enabling more Australians to access clinical trials, if interpreted through a lens of translational inclusion rather than physical enrolment [22].

Importantly, target trial emulation does not resolve limitations that may be inherent to real‐world data and cannot be addressed by analytical methods alone [20]. For example, key patient‐, clinician‐ or system‐level variables may not be collected, which could result in residual confounding and compromise any causal conclusions despite careful design [10]. Furthermore, estimates derived from administrative datasets reflect individuals for whom data are collected and do not represent members of rural subpopulations not engaged by the health system. This challenge is amplified in remote centres, where data redaction to protect patient privacy may further limit the representativeness of analyses. For these reasons, target trial emulation should be understood as a framework to strengthen causal inference about available data and not guarantee that causal inferences can be made. It should also not be viewed as a substitute for investment in the rural research capacity, data collection and clinical trial activity that could address these limitations directly.

Although target trial emulation has become increasingly adopted in other domains of medicine, to our knowledge, its application in rural health remains unexplored. English language searches of PubMed, MEDLINE, Embase, Scopus and CINAHL (2000–2025) performed using terms such as ‘target trial’, ‘emulation’ and ‘rural’ revealed no relevant results (Section S1). Yet, the approach is uniquely suited to small, distributed populations where RCTs are logistically complex [2]. Rural health services also operate across diverse contexts, varying in geography, workforce composition and service delivery models, which make traditional experimental designs difficult to implement. By enabling credible retrospective evaluation within routine service delivery, target trial emulation supports a shift from episodic prospective research to continuous learning health systems, an approach long advocated in health policy circles [23, 24].

## 3 Illustrative Example: Point‐Of‐Care Diagnostics

Rural clinicians often face uncertainty when distinguishing serious infection from self‐limiting illness, especially in facilities without onsite laboratory testing. C‐reactive protein (CRP) testing may assist in triaging patients who are suitable for local observation versus those who urgently require further investigations, but laboratory‐based testing often relies on couriers, prolonging the time to results and limiting their utility. Point‐of‐care CRP assays provide results within minutes, potentially reducing unnecessary transfers when retrieval capacity is limited [25]. An important causal question would therefore be: among patients presenting to rural hospitals, does access to point‐of‐care CRP testing reduce the rate of inter‐hospital transfer within 24 h compared with hospitals without such testing?

Because laboratory‐based CRP testing is available, withholding the point‐of‐care equivalent may be ethically inappropriate. Rather than randomising hospitals prospectively, implementation could instead occur upon hospital request, with programmatic evaluation occurring as a retrospective target trial emulation. Within this framework, eligible emergency presentations are identified from routinely collected administrative data, and hospitals are classified according to the availability of point‐of‐care CRP tests, where the primary outcome would be inter‐hospital transfer within 24 h of presentation. Analysis could then employ inverse‐probability weighting to adjust for baseline differences such as hospital size, case mix, seasonality and all other variables that could be confounders. Sensitivity analyses (e.g., falsification outcomes and negative‐control exposures) would then help evaluate the robustness of causal assumptions. Table 1 summarises how the potential trial design—the target trial—maps to its observational emulation.

**TABLE 1:** Outline of a target trial emulation of point‐of‐care (PoC) C‐reactive protein (CRP) testing and its observational emulation.

Component	Target trial specification	Observational emulation
Eligibility criteria	All emergency presentations to hospitals classified as ‘outer regional’, ‘remote’ or ‘very remote’ under the Australian Statistical Geography Standard	Same as target trial; identified retrospectively from routinely collected hospital data
Treatment strategies	Available PoC CRP vs. no PoC CRP testing (usual care)	Same as target trial: defined by the presence or absence of PoC CRP capability recorded in facility datasets
Assignment procedures	Cluster randomisation by hospital to intervention or control group	Assigned to treatment strategies based on whether patients attended hospitals with PoC CRP acquired through routine service processes
Follow‐up	From emergency presentation until 24 h post‐presentation	Same as target trial
Outcome	Inter‐hospital transfer within 24 h of presentation	Same as target trial: outcome derived from administrative and transfer records
Causal contrast	Intention‐to‐treat effect: difference in risk (risk ratio/risk difference) between patients allocated to PoC CRP‐capable hospitals vs. control hospitals	Observational analogue of intention‐to‐treat‐effect: difference in risk (risk ratio/risk difference) between patients classified into PoC CRP‐capable hospitals vs. control hospitals
Identifying assumptions	Randomisation ensures exchangeability (no unmeasured confounding)	Conditional exchangeability given measured baseline covariates; correct model specification; positivity across treatment strata
Data analysis plan	Unweighted logistic regression to estimate risk ratios and differences	Weighted logistic regression using inverse‐probability of treatment weights; sensitivity analyses with falsification outcomes and negative‐control exposures

*Note:* Although presented as a cluster design, the same causal question could be conceptualised as an individually randomised trial for alignment with the TARGET guideline.

This framing clarifies the causal question and supports transparent mapping between the hypothetical trial and real‐world data, producing timely evidence that maintains clinical equipoise while also informing broader health research and system design. Achieving transparency and reproducibility, however, requires adherence to clear reporting standards, which have not been formalised until recently.

## 4 Improving Transparency in Reporting Target Trial Emulations

The recently published 2025 TARGET guideline provides key guidance on how to report observational studies emulating a target trial [12]. This 21‐item checklist aims to improve reporting transparency, facilitate peer review and help researchers, clinicians and other readers interpret and apply the results. Although not the explicit intention, the TARGET guideline may also help researchers improve the methods and conduct of their observational analyses of interventions. In this way, the guideline could be used as an educational resource to help clarify key issues and considerations that should be addressed in target trial emulations.

Specifically, the TARGET guideline requires authors to explicitly specify each component of the target trial (eligibility criteria, treatment strategies, assignment procedures, start and end of follow‐up, outcome definitions, causal contrasts and analysis plans) and how these elements were mapped (emulated) using the real‐world observational data sources. As target trial emulations remain observational studies, the TARGET guideline encourages authors to highlight key assumptions for a causal interpretation of the results, sensitivity analyses to help assess whether these assumptions hold and the limitations of their approach. In the illustrative example described above, the key assumptions include conditional exchangeability and positive probability of each individual reaching either intervention. Making these assumptions transparent allows readers, reviewers and policymakers to understand precisely which causal question was asked and provide information on how likely it is that a causal interpretation is warranted.

In this way, application of the TARGET guideline to rural target trial emulations could strengthen the credibility and policy impact of observational evidence by building on the established language and conceptual framework of RCTs to inform causal inferences by researchers, clinicians and decision‐makers.

## 5 Conclusion

When transparently reported, target trial emulation provides rural health researchers with a rigorous yet pragmatic bridge between the ideals of RCTs and the realities of small, distributed health systems. For rural hospitals, the use of target trial emulation could transform how evidence is generated by shifting from episodic program evaluations to a continuous learning culture in which research is embedded within care through ongoing, methodologically rigorous and transparent observational analyses. Realising this vision requires sustained investment in data linkages, analytic capacity and genuine partnerships with rural communities guided by research co‐design and respect for data sovereignty. With appropriate stewardship, these tools can transform how rural evidence is translated, closing the gap between research and practice while advancing equity in rural and remote Australia.

## Author Contributions

**Tanvir Kapoor:** conceptualisation, methodology, writing (original draft), project administration. **Harrison J. Hansford:** methodology, writing (review and editing). **Brooke A. Spaeth:** conceptualisation, writing (review and editing). **Adam D. Irwin:** supervision, writing (review and editing). **Aidan G. Cashin:** methodology, supervision, writing (review and editing).

## Funding

Harrison J. Hansford was supported by a Centre for Pain IMPACT Postgraduate Scholarship and a Neuroscience Research Australia PhD Pearl sponsored by Sandra Salteri. Adam D. Irwin is supported by an Australian National Health and Medical Research Council (NHMRC) Investigator Grant (ID 1197743). Aidan G. Cashin was supported by an Australian NHMRC Investigator Grant (ID 2010088).

## Disclosure

Provenance: Not commissioned; externally peer reviewed.

## Conflicts of Interest

Adam D. Irwin has received honoraria for speaking from Biomerieux and Gilead which were paid to his institution.

## Supporting information


**Data S1:** mja270205‐sup‐0001‐supinfo.docx.

## Data Availability

The authors have nothing to report.
