# Supplementary material for: Target Trial Emulation and the TARGET Guideline to Advance Rural and Remote Health Research
Source: Med J Aust. 2026 May 14;224:e70205. doi: 10.5694/mja2.70205 (PMC13172931; doi:10.5694/mja2.70205)
Supplement: Supplementary file 1 — Data S1: mja270205‐sup‐0001‐supinfo.docx. [file MJA2-224-0-s001.docx]

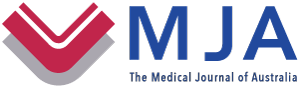


Supporting Information

Supplementary material

**This appendix was part of the submitted manuscript and has been peer reviewed.
It is posted as supplied by the authors.**

Appendix to: T. Kapoor, H. J. Hansford, B. A. Spaeth, A. D. Irwin, and A. G. Cashin. Target Trial Emulation and the TARGET Guideline to Advance Rural and Remote Health Research. *Med J Aust* 2026; doi: 10.5694/mja2.70205.

**SECTION S1. Search Strategy**

PubMed (incorporating MEDLINE) was searched for

(“target trial*” OR “trial emulation” OR “emulated trial*” OR “targeted trial*” OR “emulat* randomi*ed” OR “emulat* design”) AND (“rural” OR “remote” OR “regional” OR “rural health” OR “rural hospital*” OR “rural population*” OR “rural health services” OR “remote communit*”)

Embase was searched for

(“target trial” OR “trial emulation” OR “emulated randomi?ed” OR “causal inference”) AND (rural OR rural health OR rural population OR rural area OR rural hospital OR regional OR remote)

Scopus was searched for

(“rural” OR “remote” OR “regional”) AND (“target trial” OR “trial emulation” OR “causal inference”) AND (“health” OR “health services” OR “policy”)

CINAHL was searched for

(“rural” OR “remote” OR “regional”) AND (“target trial” OR “trial emulation” OR “causal inference”) AND (“health” OR “healthcare” OR “health services”)

The search period was 1 January 2000 to 30 September 2025 and was limited to human studies published in English. Searches were supplemented with targeted Google Scholar queries using combinations of “target trial emulation rural health.” Reference lists of key methodological and applied papers were screened to identify additional relevant studies.
